# New insights into aging-associated characteristics of female subcutaneous adipose tissue through integrative analysis of multi-omics data

**DOI:** 10.1080/21655979.2021.2020467

**Published:** 2022-01-09

**Authors:** Zichao Li, Shun Wang, Shaojie Liu, Ziwen Xu, Xiaowei Yi, Hongtao Wang, Juanli Dang, Xinxin Wei, Bingyue Feng, Zinuo Liu, Ming Zhao, Qiong Wu, Dahai Hu

**Affiliations:** a Department of Burns and Cutaneous Surgery, Xijing Hospital, Fourth Military Medical University, Xi’an, Shaanxi, China; b BD Life Science (Guangzhou) Co., Guangzhou Laidi Innovation and Technology Park, Guangzhou, Guangdong, China; c Department of Urology, Xijing Hospital, Fourth Military Medical University, Xi’an, Shaanxi, China; d Department of Basic Medicine, Fourth Military Medical University, Xi’an, Shaanxi, China; e West China School of Public Health and West China Fourth Hospital, Sichuan University, Chengdu, Sichuan, China; f Department of Plastic and Reconstructive Surgery, Xijing Hospital, Fourth Military Medical University, Xi’an, Shaanxi, China; g School of Stomatology, Jiamusi University, Jiamusi, Heilongjiang, China; h The First Hospital of Xi’an, Xi’an, Shaanxi, China; i Provincial Key Laboratory of Biotechnology of Shaanxi, Key Laboratory of Resource Biology and Modern Biotechnology in Western China, Faculty of Life Science, Northwest University, Xi’an, Shaanxi, China

**Keywords:** Subcutaneous adipose tissue, aging, obesity, multi-omics profiling, tissue repair and reconstruction

## Abstract

Aging could be critical in limiting the application of subcutaneous adipose tissue (SAT) in tissue repair and reconstruction. However, no systematic study on the characteristics of SAT aging has been conducted. In this study, a scanning electronic microscope was used to detect the structural and compositional changes of SAT collected from nine females in three age groups. Multi-omics data of SAT from 37 females were obtained from Gene Expression Omnibus database, and 1860 genes, 56 miRNAs, and 332 methylated genes were identified as being differentially expressed during aging among non-obese females. Using Weighted Correlation Network Analysis (WGCNA), 1754 DEGs were defined as aging-associated genes for non-obese females, distributed among ten co-expression modules. Through Gene Ontology enrichment analysis and Gene Set enrichment analysis on those aging-associated DEGs, SAT aging was observed to be characterized by variations in immune and inflammatory states, mitochondria, lipid and carbohydrate metabolism, and regulation of vascular development. SUPV3L1, OGT, and ARPC1B were identified as conserved and core SAT-aging-related genes, as verified by RT-qPCR among 18 samples in different age groups. Multi-omics regulatory networks of core aging-associated biological processes of SAT were also constructed. Based on WGCNA, we performed differential co-expression analysis to unveil the differences in aging-related co-expression patterns between obese and non-obese females and determined that obesity could be an important accelerating factor in aging processes. Our work provides a landscape of SAT aging, which could be helpful for further research in fields such as repair and reconstruction as well as aging.

## Introduction

Subcutaneous adipose tissue (SAT), an ideal autologous soft tissue filler with no immune rejection [1], plays an important role in the repair and reconstruction of tissues and in cosmetic surgery [2,3]. Aging is associated with progressive changes and functional declines at the organismal, tissue, and cellular levels [4,5]. Previous studies found that the aging-related alteration of physiological state and metabolic patterns of adipose tissue potentially leads to some adverse reactions, such as chronic skin ulcer, scar formation, and skin carcinoma [6]. As age advances, fat redistribution is usually accompanied with the diminishing of the SAT of the lower limbs and increase of visceral adipose tissue (VAT) and ectopic fat, which induces the metabolic syndrome [7]. On the other hand, the aging process has always been associated with cellular senescence, the decrease and dysfunction of precursor adipocytes, and a lipotoxic state with accumulation of free fatty acids in intercellular matrix [8,9].

Substantial evidence has proved that obesity could considerably accelerate aging and affect cellular and molecular processes in a fashion resembling aging [10,11]. The proportion of obese adults, particularly among middle-aged population and elders, is increasing rapidly worldwide. Thus, obesity among them has become a major public health concern [12]. SAT stores about the majority of systemic fat; thus, the excessive accumulation of white adipose in SAT may contribute the most to patient obesity and accompany the aging process, particularly in the middle age. However, in the elder, losing of SAT could also be detected with decreased somatic function and poor health during aging. Clinically, obesity increases the risk of aging-associated delayed wound healing and limits the application of autologous fat transplantation because of the dysfunction of adipose tissue [13]. However, the mechanisms of these clinical manifestations have not been systematically clarified to be caused by the SAT aging process.

SAT could experience the specific aging-related variation during the systematic aging process. We aim to describe the changes of the composition and structure of SAT in different age groups through the scanning electron microscopy. More importantly, comprehensive integrative analysis of multi-omics data of mRNA, miRNA, and methylation data of SAT were performed to unveil the biological changes, key molecules and potential influence factor (obesity) in SAT aging. Our work would systematically expound the aging-related variation of composition, structure, and biological characteristics of SAT, which provide a better understanding of specific disorders and reduced clinical application in tissue reconstruction associated with SAT aging. Our research discussed the potential of SAT on exploring the connection between obesity and human aging.

## METHODS

### Scanning electron microscopy (SEM)

SATs were obtained from patient abdominal flaps that were to be discarded at Xijing Hospital (Xi’an, China). Three biological repeats in each group were included for SEM and their clinic information was shown in Table S1. Informed consent regarding the purpose and procedure of the study was completed from volunteer patients. This study was approved by the Ethics Committee of Xijing Hospital, Fourth Military Medical University. The adipose tissue was washed with ice-cold PBS thrice. The SAT were fixed with 2% glutaraldehyde and then 1% osmium tetroxide for 1 hour. SEM at Servicebio Co., Ltd (Wuhan, China) was used to observe the morphology of SAT. Diameters and cell numbers of adipocytes were quantified using ImageJ software and analyzed using Prism 8. Blinding measures for the collagen morphology were subjective and the 5 observers after training were included. The evaluation parameters and corresponding scores were listed in the Table S2, and the obtained score from observers were averaged for analysis.

### Data sources

The raw sequencing data of mRNA (GSE25401), miRNA (GSE25470), and methylation (GSE24884) datasets were downloaded from GEO. On basis of the annotation of clinical information through the datasets, the involved 37 female in our study were grouped by age (Young, 20–30 years old; Middle-aged, 40–50 years old; Elderly: ≥60 years old) and obesity status (non-obese and obese) as presented in Table S3 and S4. The original research which was the source of our sequencing and clinical data, defined that BMI>30 kg/m2 should be considered as obesity, and the BMI of those obese population is 40.9 ± 1.3 kg/m2 and 24.1 ± 1.8 kg/m2 for non-obese population [14]. All samples were investigated in the morning after an overnight fasting and their weight was in a stable status prior to sample collection.

### Integrative analysis of multi-omics data

Aging spectrums were constructed on the basis of differentially expressed mRNAs, miRNAs, and methylated genes. The probe IDs in microarray expression profile were converted to gene symbol through matching the Hugo gene symbol. When calculating the gene expression levels, probes belong to more than one gene were excluded and if multiple probes were marked corresponding to one gene, only the probe had highest signal strength among all samples on average was reserved. The expression levels of microRNAs were directly exported from miRNA microarray data. HumanMethylation27k BeadChip was used for identifying the DNA methylation level. Each gene was mostly corresponding to two methylation locus, and methylation levels were defined by averaging the testing values of those two methylation locus. Gene Set Enrichment Analysis (GSEA) and Gene Ontology (GO) enrichment analyses of the selected differentially expressed genes (DEGs) in different groups were performed to identify the aging-related biological processes of SAT using the *clusterProfiler* package [15,16]. Protein–protein interaction (PPI) analysis via the STRING dataset was used to construct a co-expression network and regulatory networks between aging-related DEGs [17]. Network node colors corresponding to co-expression modules were assigned. Targeted prediction of interaction relationships between differentially expressed miRNAs (DEMs) and DEGs was obtained from mirWalk2 through the binding sites of the 3ʹUTRs [18].

### Weighted gene co-expression network analysis (WGCNA)

DEGs between various age groups of obese and non-obese samples were united and defined as the candidate aging-related DEGs of SAT for further constructing a scale-free gene co-expression network. Aging-related genes were grouped into modules via hierarchical average linkage clustering. The signed correlation method was used to transform the correlation coefficient of co-expression between nodes using the expression adjacency (between nodes) = (1+ correlation)/2, which weakened negative correlations and enhanced positive ones. The soft thresholding power (β) was set to 14 to generate an appropriate scale-free co-expression network. Clustering genes hierarchically used the dynamic tree cut method with the dissimilarity matrix (1-TOM) [19]. The minimum cluster size required 50 genes. The clustered modules with high similarity were merged with a height cutoff of 0.4.

### Differential gene co-expression analysis

To quantify the differences in aging-related co-expression patterns in SAT-aging between non-obese and obese females, we performed differential co-expression (DiffCoEx) analysis based on WGCNA [20]. Adjacency matrices for aging-related DEGs of non-obese and obese cases were calculated separately and adjacency values between nodes were defined as follows: if the correlations coefficient was >0, adjacent values equaled the correlation coefficient; if correlations coefficient was ≤0, adjacent values were set as 0. The topological overlap matrix was derived from the matrix of differences in powered adjacencies between non-obese and obese cases and used as a distance metric. Genes were clustered using average hierarchical clustering, so gene co-expression modules were partitioned with unique and similar variation patterns between the two populations. DiffCoEx modules were further identified using the hybrid method of dynamic tree cutting. The hierarchical clustering tree was cut at a height of 0.94. The minimum cluster size of each module was 20 and merged cut height was ≤0.2.

### RT-qPCR analysis

SATs from 18 volunteers were used for RT-qPCR analysis, which were divided into 3 age groups and each group include 6 females. The clinic information of the volunteers was shown in Table S1. Total RNA was extracted using the TRIzol reagent and reverse transcription (RT) was performed using PrimeScript™ RT Master Mix (TaKaRa, Japan; #RR036A). Finally, qPCR was performed using TB Green® Premix Ex Taq^TM^ II (TaKaRa, #RR820A). Primers for qPCR are listed in Table 1. All data are expressed as the mean standard deviation of at least three independent experiments. Before the analysis, the quantitative data were tested for normality and homogeneity of variance using GraphPad Prism 8. Statistical analyses were performed using Student’s test or linear correlation. Differences were considered significant when p < 0.05.

**Table 1.** Primers used for RT-qPCR analysis

Target	Forward Primer	Reverse Primer
*SUPV3L1*	TGCTGATTATGGACTTGATGCTC	CCACATCCAGGGAATGAGACT
*OGT*	TCCTGATTTGTACTGTGTTCGC	AAGCTACTGCAAAGTTCGGTT
ARPC1B	*CAAGGACCGCACCCAGATT*	*TGCCGCAGGTCACAATACG*

### Statistics and visualization

Most statistical analyses were performed using R 4.0.5. The RankProd package was used to identify DEGs and DEMs between groups [21], and *limma* package was used to identify differentially methylated genes (DMGs) with differential mean methylation levels ≥5% between groups [22]. Spearman correlation coefficient <−0.5 indicated the existence of regulatory relationship of aging-related expression patterns of DEMs–DEGs and DEGs–DMGs. The *WGCNA* package was used for co-expression network analysis [23]. DEGs, DEMs, and DMGs were visualized using heatmaps. CytoScape software was used to construct the interaction network [24]. *P* ≤ 0.05 was set as the cutoff threshold.

## Results

Our findings could be divided into three parts. First, the compositional and structural changes of SAT in aging process could be qualitatively or quantitatively evaluated with SEM. Then, WGCNA method was performed to identify aging-associated DEGs, their co-expression patterns, conserved functional genes and core biological variation. Integrating the multi-omics aging profiles, the multi-omics interaction relationships of the SAT-aging-related core BP were demonstrated clearly for non-obese female. Finally, DiffCoEx analysis based on WGCNA could unveil the difference between non-obese and obese on influence for the SAT aging process in each age group. Therefore, the landscape of the SAT aging could be detailed on multiple levels.

### Variation patterns of adipocyte and collagen morphology in SAT during aging

**Figure 1.**
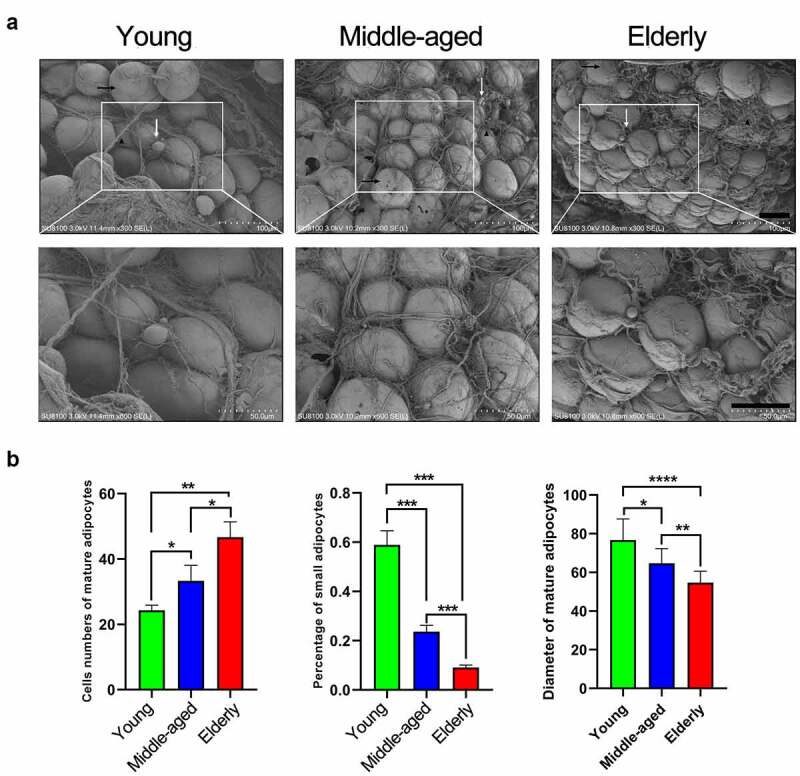
Morphology and quantification of adipocytes and collagen of SAT during aging. (a) SEM images of SAT in different age groups. *Scale bar* = 100 μm or 50 μm. (b) Numbers of the mature adipocytes, percentage of small adipocytes and diameter of mature adipocytes in different age groups in the same-sized fields.

As shown by SEM, adipocytes and collagen morphology in SAT varied morphologically during aging (Figure 1(a)). SAT aging was accompanied by a decreasing quantity of collagen and the morphological variation of collagen was characterized by its aggregating into bundles, curling, wrinkling, and breaking (Table 2). Cell counts of mature adipocytes and the percentage of the small adipocytes were quantified in different age groups with the same area of view in Figure 1. The cell numbers of mature adipocytes in the targeted view were 24.33 ± 3.795 in Youth, 33.33 ± 11.74 in Middle age, and 46.67 ± 11.736 in Elderly, and the corresponding percentages of small adipocytes were 59%±14%, 24%±6%, and 9%±3% respectively. The size of the mature adipocytes of SAT also declined with the aging process with 76.66 ± 10.96 μm in Youth, 64.72 ± 7.54 μm in Middle age, and 54.72 ± 5.92 μm in Elderly (Figure 1(b), Table 3).

**Table 2.** Blinding measures for the morphology of collagen and diameters of adipocytes of SAT. Scores from five observers were demonstrated as mean ± standard of the nine non-obese samples on basis of SEM images. Sample 1–3 were Youth; 4–6 were Middle-age and 7–9 were Elder

Group	Collagen curling	Collagen wrinkling	NSC integrity	NSC density	Bundle formation of collagen	Diameter of the mature adipocytes (μm)
Sample1	1.6 ± 0.54	1.8 ± 0.44	4.6 ± 0.54	2.75 ± 0.5	2.6 ± 0.54	77.5 ± 13.91
Sample 2	1.4 ± 0.54	1.6 ± 0.54	4.6 ± 0.54	2.5 ± 0.57	2.8 ± 0.44	83.33 ± 5.77
Sample 3	1.2 ± 0.44	1.4 ± 0.54	4.2 ± 0.44	2.75 ± 0.95	2.6 ± 0.54	69.16 ± 10.1
Sample 4	3.2 ± 0.44	3 ± 0.7	3.6 ± 0.54	3.25 ± 0.5	4 ± 0	60 ± 5
Sample 5	3.2 ± 0.44	2.8 ± 0.44	3.4 ± 0.89	2.75 ± 0.5	3.8 ± 0.44	63.33 ± 2.88
Sample 6	3.6 ± 0.54	3.2 ± 0.83	3.6 ± 0.54	3.25 ± 0.5	3.2 ± 0.44	70.83 ± 10.1
Sample 7	4.8 ± 0.44	4.2 ± 0.44	1.4 ± 0.54	3.25 ± 0.5	4.8 ± 0.44	51.66 ± 7.63
Sample 8	4.8 ± 0.44	4.6 ± 0.54	1.4 ± 0.54	2.5 ± 1	4.8 ± 0.44	59.16 ± 5.2
Sample 9	4.6 ± 0.54	4.2 ± 0.44	2.2 ± 0.44	4 ± 0.81	4.6 ± 0.54	53.33 ± 2.88

**Table 3.** Statistical analysis for the scores of the morphology of collagen and mature adipocytes in SEM images of samples within different age groups

Group	Young	Middle-aged	Elderly	P-value
Collagen curling	1.4 ± 0.5	3.33 ± 0.48	4.73 ± 0.45	*P* < 0.0001
Collagen wrinkling	1.6 ± 0.5	3 ± 0.65	4.33 ± 0.48	*P* < 0.0001
NSC integrity	4.46 ± 0.51	3.53 ± 0.63	1.66 ± 0.61	*P* < 0.0001
NSC density	2.33 ± 0.61	3.4 ± 0.63	4.6 ± 0.5	*P* = 0.0602
Bundle formation of collagen	2.66 ± 0.48	3.66 ± 0.48	4.73 ± 0.45	*P* < 0.0001
Diameter of the mature adipocytes (μm)	76.66 ± 10.96	64.72 ± 7.54	54.72 ± 5.92	*p* < 0.0001

### Integrative analysis of multi-omics aging profiles of SAT

**Figure 2.**
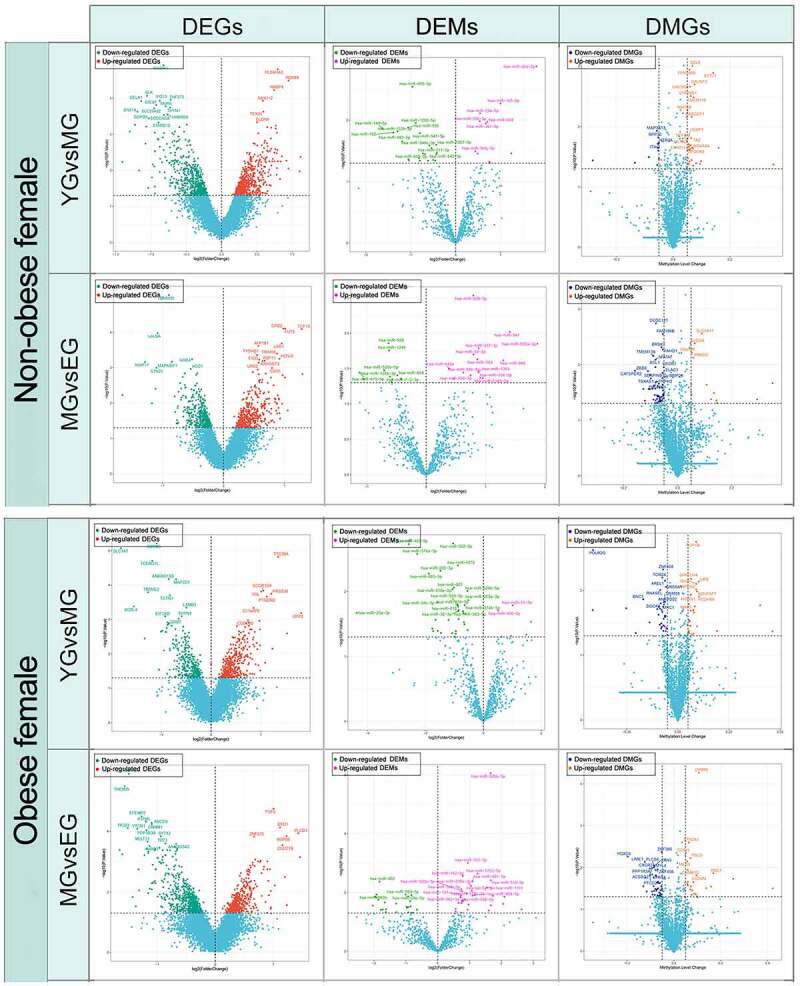
Differentially expressed mRNAs, miRNAs, and methylated genes in aging processes for obese and non-obese females.

Abbreviations: DEGs, differentially expressed genes; DEMs, differentially expressed miRNAs; DMGs, differentially methylated genes; YG, Youth group; MG, Middle-aged group; EG, Elderly group.

The multi-omics expression profiling data from 37 females in Table S3 demonstrated significant age-dependent variations in mRNA, miRNA, and methylated genes during SAT aging. 1860 DEGs, 56 DEMs, and 332 DMGs were detected between the age groups in non-obese cases, and 1623 DEGs, 95 DEMs, and 348 DMGSs were detected in obese cases (Figure 2). Further integrative analysis between DMGs-DEGs–DEMs were performed based on multi-omics aging profiles of the non-obese population to elucidate the aging-associated molecular interaction patterns of SAT comprehensively. The DEGs and DEMs in Figure S1 demonstrate rich regulatory relationships, with 500 regulatory relationships of moderate correlation intensity (r <−0.5, p < 0.05). Despite methylated genes being identified during SAT aging in non-obese cases (Sheet S1), none of those genes were SAT-aging-related DEGs (p > 0.05). Those methylated genes were mainly enriched in molecular functions, cell signals and communication, cellular response, and metabolism among obese and non-obese females (Figure S2).

### Non-obese SAT aging-related core molecular regulation and biological processes

**Figure 3.**
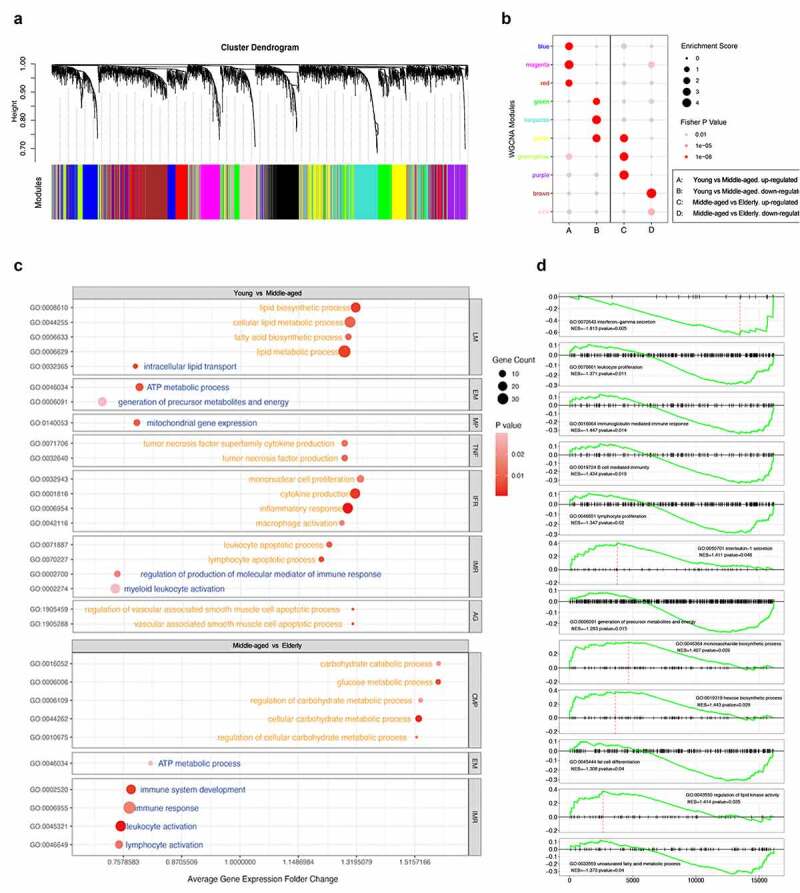
The co-expression patterns of genes and visualization of core GOBP pathways of SAT in aging process. (a) Visualization of tree diagram and co-expression patterns of genes based on the adjacency-based dissimilarity of the hierarchical clustering genes. (b) 10 aging-related gene co-expression modules with different enrichment status in each group. Enrichment Score indicated the ratio of DEGs in the specific module to all genes involved in the WGCNA network. (c) GOBP enrichment analysis for selected DEGs. (d) Gene set enrichment analysis of genes based on age groups. Normalized enrichment score (NES) was presented in chart. *P* Value < 0.05 was considered significant.

**Figure 4.**
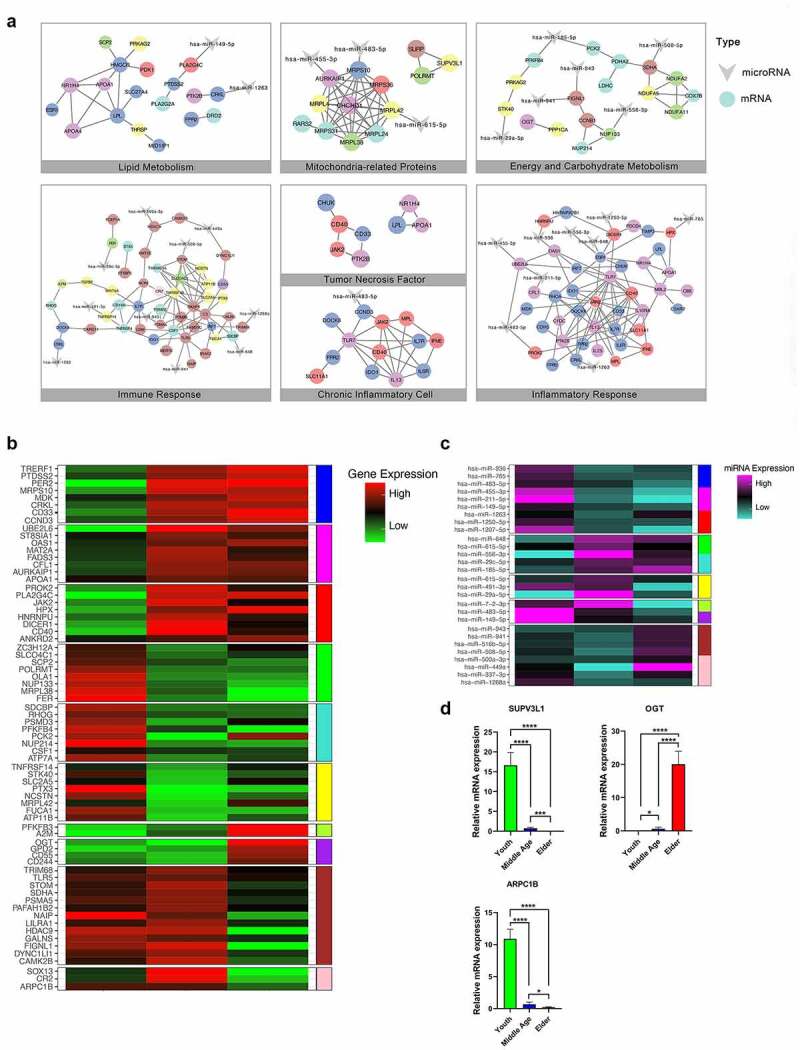
The regulatory networks between DEGs and DEMs and RT-qPCR. (a) The regulatory networks of DEGs-DEMs involved in 7 core functional subnetwork in SAT agingbased on WGCNA. (b,c) Heatmaps of DEGs and DEMs involved in the 7 regulatory networks. DEGs and DEMs were distributed in different co-expression modules with different colors. (d) Quantitative analysis of the RT-qPCR assays for SAT.

Among 4560 aging-related candidate genes of SAT presented in Figure S3A, 11 aging-related co-expression modules including 1754 DEGs were identified in Table S2, which were considered closely associated with the aging process via hierarchical average linkage clustering (Figure 3(a,b), Figure S3B) (Table S5). A soft-thresholding power of 14 was set for network construction (Figure S3C-D). On this basis, aging-related biological processes (BP) of SAT were identified in Figure 3(c,d) using GO and GSEA methods. The changes of BP associated with SAT aging focused on inflammatory states, immune response, mitochondrial gene expression, tumor necrosis factor (TNF), lipid metabolism, energy and carbohydrate metabolism, and regulation of vascular development. Accordingly, seven functional interaction subnetworks were further constructed to demonstrate the regulatory relationship of the core aging-related BP of SAT (Figure 4(a)), which involved 195 core DEGs in different co-expression modules as well as 70 core DEGs and 28 core DEMs with existing interaction relationships shown in Figure 4(b,c). DEGs in blue, red, and magenta modules had relatively more interactions in the network. On basis of the aging associated DEGs involved in the core functional pathways related to SAT metabolism, we demonstrated the shifts in expression of the metabolic gene clusters on carbohydrate metabolism, energy metabolism and lipid metabolism in different age groups in Figure S4.

### Conserved functional SAT aging-related genes

Based on the SAT aging-related gene expression profiles of obese and non-obese females, we identified three aging-related DEGs (O-linked N-acetylglucosamine (GlcNAc) transferase (OGT), Suv3 like RNA helicase (SUPV3L1), and actin-related protein 2/3 complex subunit 1B (ARPC1B) that could be considered conserved potentially functional genes between both populations. This was validated via RT-qPCR and the three genes showed a consistent trend in SAT aging. RT-qPCR analysis showed that the mRNA level of *OGT* significantly increased and *SUPV3L1* declined with aging (p < 0.05). *ARPC1B* mRNA expression virtually decreased in Middle-aged and Elderly groups (p < 0.05, Figure 4(d)). These three conserved genes in SAT participate in important aging-related GOBP pathways that could influence the outcome of tissue repair considerably.

### Different aging patterns of SAT between obese and non-obese females

**Figure 5.**
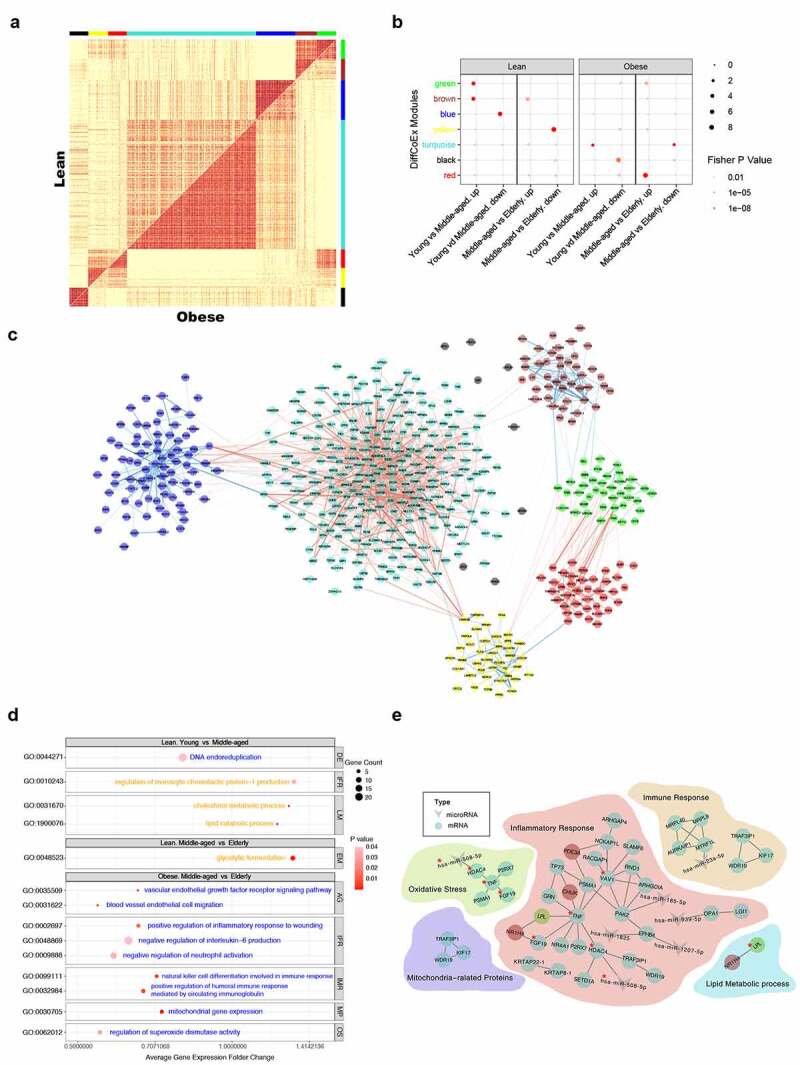
DiffCoEx analysis based on aging related genes of SAT between obese and non-obese female. (a) The comparative correlation heatmap showed the differentially co-expressed modules of SAT between obese and non-obese female based on aging related genes. The upper diagonal of the main matrix showed a correlation between pairs of genes among the non-obese female. The lower diagonal showed a correlation between the same gene pairs among the obese female. Color bars represented the differentially co-expressed modules. (b) 7 DiffCoEx modules with different enrichment status of SAT in each group. Enrichment Score indicated the ratio of DEGs in a specific module to all genes involved in the DiffCoEx network. (c) Differential co-expression network of SAT between obese and non-obese female. Colors represented the DiffCoEx modules. Red lines showed stronger correlation in obese female but blue lines in non-obese female. The thickness represented the correlation strength. (d) Bubble plots foraging related GOBP enrichment pathways. (e) The regulatory networks of DEGs-DEMs involved in the aging associated biological processes based on DiffCoEx analysis of aging process between obese and non-obese female. ‘*’ represented the potentially core molecules in the regulatory networks of obese female according to DiffCoEx analysis. *P* Value < 0.05 was considered significant.

DiffCoEx analysis was performed to explore the differences in the gene expression profiles of SAT aging between obese and non-obese females. Of the 4560 aging-related genes, 539 DEGs were assigned to one of seven modules with different co-expression patterns in different aging stages between obese and non-obese females (Figure 5(a,b)). We found that 202 genes in green, blue, brown, and yellow modules had more significant co-expressed patterns with SAT aging in non-obese females. The red, black, and turquoise modules, containing 337 genes, were notably co-expressed during aging in the obese group (Table S6). The differential co-expression gene network is visualized in Figure 5(c). GO enrichment analysis was also performed for specific co-expression modules in each group to reveal the different aging-related BP of SAT between obese and non-obese females. Accordingly, aging-related enriched BP pathways in non-obese females were mainly detected from Young to Middle-aged, while for obese females, the enriched BP mostly presented in progression from Middle-aged to Elderly (Figure 5(d)). The SAT aging process of non-obese females from Young to Middle-aged was always characterized in the accumulated monocyte chemotactic protein-1 (MCP-1), up-regulated lipid metabolism relevant pathways, and down-regulated DNA duplication pathways. Later, upregulated glycolytic fermentation was highly enriched during the process from Middle-aged to Elderly. Compared with non-obese cases, the SAT of obese cases were likely to experience different aging processes. From Middle-aged to Elderly, the enriched BP pathways in obesity was associated with the increased inflammatory mediators, suppressed immune response and decreased mitochondrial gene expression, regulation of wounding, vascular endothelial cell growth, and superoxide dismutase activity (Figure 5(d)). Furthermore, Figure 5(e) demonstrated the mRNA–miRNAs regulatory networks for obesity-specific aging-related BP compared with non-obese cases.

## Discussion

SAT is a type of widely distributed adipose tissue in human body. However, to our knowledge, no systematic work has been performed to expound the aging-related characteristics of SAT. Thus, through exploring the alteration patterns of SAT during aging, some tough clinical problems and phenotypes could be potentially explained in the future.

As the main filling material under the skin layer, SAT undergoes significant phenotypic variations in composition and structure and brings about obvious changes in appearance during aging. Most researches confirmed that small adipocytes diameter between 7 and 20 μm were considered and the diameter of those mature adipocytes were usually >60 μm [25]. Consistent with Donato’s study [26], we confirmed that mature adipocytes of SAT gradually got smaller and counts of small adipocytes decreased from the stages of Young to Elderly. Mature adipocytes are mainly responsible for storing triglycerides and a large part of small adipocytes are preadipocytes, and the decrease of small adipocytes during SAT aging would limit the quality of mature adipocytes through the reduction of the proliferative and differentiative capacities of preadipocytes. The changes of collagen morphology would reduce the mechanical properties of SAT as aging progressed [27]. The weakened supporting capacity of SAT in elders might influence structural instability and application in several ways, such as the development of quiescent wrinkles and regional dent, and limits the clinical application of SAT in soft tissue filling.

On the basis of the aging spectra of SAT along with multi-omics data, we found that methylated genes had little influence on the expression of aging-related DEGs during SAT aging in obese and non-obese females. Combining the WGCNA with the enrichment analysis, 195 core DEGs were reserved in our focused aging-associated biological regulatory subnetworks. Results implied that the immune status of SAT was distinct in different age groups. The changes of immune and inflammatory status might partly attribute to apoptosis and decreased activation of lymphocytes and leukocytes, and increased inflammatory cytokines and cells in middle-aged group and decreased activation of lymphocytes and leukocytes and immunity in Elderly. The infiltration of inflammatory cytokine and cells especially macrophages, were reported to promote the development of severe fibrosis and contribute to a higher risk of formation of hypertrophic scars during the wound healing process [28]. TNF production and reduced mitochondrial gene expression could be factors of regional insulin resistance [29,30], which would inhibit angiogenesis, reorganization of elastic fibers, and wound healing during the aging process reported by previous studies [31].

Shifts in the metabolic gene clusters and metabolic patterns were firstly demonstrated in our work. A transition of dominant metabolism from lipid metabolism to carbohydrate metabolism was presented from Middle age to Elderly. Increased lipid and fatty acid biosynthesis as well as reduced lipid transport in Middle age might synergistically contribute to excessive deposition of abdominal SAT. Whereas upregulated carbohydrate metabolism was related to the local tissue energy supply mode in elderly that the shift in substrate oxidation was caused by age-related changes in skeletal muscle respiratory capacity [32]. Furthermore, the decreased vascular smooth muscle cell apoptosis could significantly influence the regulation of vascular development in Elder. Aging-associated chronic inflammation state and defective angiogenesis would be potential targets to improve the fat graft survival after autologous fat transplantation and other clinical application in Elder [33].

DiffCoEx analysis implied that obesity is a potential key factor that affects the SAT aging process and might be related to many adverse events in tissue repair and reconstruction clinically [34]. By comparison, the occurrence of some specific aging-related biological processes of non-obese population were mainly from Young to Middle age, while the specific aging-related variations for obese females were concentrated in Middle age to Elder (Figure 5(d)). Aging-related production of inflammatory mediators and decreased immune states were aggravated for the obesity of SAT. In obese females, the suppression of natural killer (NK) cells and the biological activity of immunoglobulin in Elder were significantly related to human nonspecific immunity. The downregulated differentiation of NK cells would influence their normal biological function and the functionally impaired NK cells were reported to decrease the cellular activity and maturation of stem cells, further impairing the regeneration of damaged tissues [35]. Down-regulated HDAC4, VAV1, and TNF expression were likely to be another important factors increasing the inflammation status of SAT in obese people, which were related to the negatively regulating the activation and development of T and B cells [36]. Furthermore, down-regulated wound healing-related inflammatory responses pathways and activity of vascular endothelial cell may cooperate to be associated with the higher risk of chronic wounds and lower rates of angiogenesis in fat graft for obese elders [37].

SUPV3L1, OGT, and ARPC1B were identified as the conserved aging-related functional genes for both obese as well as non-obese females in our research, and they were verified to be differentially expressed by an RT-qPCR assay. In previous studies, disruption of SUPV3L1 displayed growth retardation and aging phenotypes, including loss of adipose tissue mass and atrophy of the dermis and SAT, which also was a potential marker of aging [38]. ARPC1B was a functional gene associated with immunodeficient and inflammatory states [39,40], so the down-regulated ARPC1B in the aging process could be related to the regional chronic inflammatory status and delayed tissue repair in SAT aging. Besides, overexpression of OGT was also reported to cause delayed wound healing in patients with skin ulcers, which prevents the keratinocytes at the wound margin from migrating into the wound to promote re-epithelialization [41]. Therefore, these three genes could be the potential targets to delay the senescence of SAT and make an improvement in tissue repair and reconstruction.

Compared with SAT aging process in non-obese females, aging-associated metabolic variations were less obvious for obese females according to DiffCoEx analysis. With accumulation of SAT in Middle Age, the up-regulated cholesterol metabolism and lipid catabolic process for non-obese females could partly counteract the excessive SAT to reach a healthy and stable state. For Elder, aging process of SAT was accompanied with the down-regulated ATP metabolic pathways due to the cellular senescence, the increased carbohydrate-related metabolism, such as glycolytic fermentation, might be one of the compensatory mechanisms for ATP supply among non-obese elders. Besides, increased adipose tissue-derived fibroblast growth factor and decreased FGF19 and mitochondrial gene expression were especially detected in SAT aging for obese females, which were reported associated with aging-related variation in endocrine and metabolism regionally [42]. Meanwhile, due to the down-regulated superoxide dismutase activity for obese females, the oxidative stress might be increased which could lead to accelerated SAT aging process and mediate regional insulin resistance from Middle aged to Elder [43,44]. These findings could potentially explain why elders, particularly obese ones, are more likely to form chronic wounds.

Previous studies demonstrated that systemic aging was accompanied by the alteration of immune status, inflammation, and angiogenesis [45,46], partly in accordance with our findings on SAT aging. However, some aging-associated features were unique to SAT, such as metabolic variations, which may improve our understanding of diversified aging process systemically. Given the wide distribution and our findings, SAT could be considered as a novel ideal research object to explore lipid metabolism disorders and aging.

Our study has the following limitations. First, all the multi-omics expression profiling data used were obtained from GEO database. Given that no research fully matched our direction of exploration, only partial sections were selected for further exploration, so no other available datasets could be obtained for further validation. Second, sampling was limited to abdominal SAT and all the samples in our research were obtained from females. Third, we did not uncover strong evidence that genes in our focused biological processes were regulated by methylation, and only the interaction network between genes and miRNAs was demonstrated. Despite these limitations, the reliable data sources, appropriate statistical models, and experimental verification ensure the reliability of our conclusions.

## Conclusion

The morphology and quantities of collagen and adipocytes were significantly influenced by aging of SAT. The aging-related DEGs identified by WGCNA contributed to the variations in some specific biological processes related to immune and inflammatory states, mitochondria, and lipid and carbohydrate metabolism. SUPV3L1, OGT, and ARPC1B were then verified potentially conserved target DEGs of SAT aging. DiffCoEx analysis revealed that obesity could be regarded as an accelerating factor for SAT aging with different variation patterns. Above all, aging-related variations of composition, structure, biological processes, multi-omics regulatory networks, and core conserved genes of SAT were expounded comprehensively for the first time.

## Supplementary Material

Supplemental Material
