# Supplementary material for: New insights into aging-associated characteristics of female subcutaneous adipose tissue through integrative analysis of multi-omics data
Source: Bioengineered. 2022 Jan 9;13(2):2044–57. doi: 10.1080/21655979.2021.2020467 (PMC8973830; doi:10.1080/21655979.2021.2020467)
Supplement: Supplemental Material [file KBIE_A_2020467_SM1083.zip › supplementary/Supplementary Figure legendsclean.docx]

**Supplementary figure legends**

**Figure S1.** Heat map of DEMs(A) and DMGs(B) in the aging spectrum of multi-omics regulatory network.

**Figure S2.** Functional enrichments of gene methylation in non-obese (A) and obese groups (B).

**Figure S3.** Analysis of the network topology for adjacency matrix weighting parameters (power). A. 4560 genes united by the 10 parts were considered as the potential aging-related genes for WGCNA. B. Hierarchical average linkage clustering. Branches of the dendrogram represent genes with similar expression patterns. C and D. The x-axis represents soft threshold (power), and the y-axis represents the scale free fitting index and connectivity for each power. The soft-thresholding power for network construction was set at 14.

**Figure S4.** The metabolic gene clusters on carbohydrate metabolism, energy metabolism and lipid metabolism in different age groups.
