# Supplementary material for: New insights into aging-associated characteristics of female subcutaneous adipose tissue through integrative analysis of multi-omics data
Source: Bioengineered. 2022 Jan 9;13(2):2044–57. doi: 10.1080/21655979.2021.2020467 (PMC8973830; doi:10.1080/21655979.2021.2020467)
Supplement: Supplemental Material [file KBIE_A_2020467_SM1083.zip › supplementary/Table S1clean.docx]

| **Sample ID** | **Gender** | **Age** | **Height (m)** | **Weight (kg)** | **BMI (kg/m^2^)** | **SEM** | **RT-qPCR** |
| --- | --- | --- | --- | --- | --- | --- | --- |
| 1 | female | 23 | 1.70 | 58 | 20.069 | Y | Y |
| 2 | female | 24 | 1.64 | 57 | 21.193 | Y | Y |
| 3 | female | 26 | 1.68 | 52 | 18.424 | Y | Y |
| 4 | female | 28 | 1.66 | 64 | 23.225 |  | Y |
| 5 | female | 21 | 1.65 | 56 | 20.569 |  | Y |
| 6 | female | 23 | 1.68 | 56 | 19.841 |  | Y |
| 7 | female | 48 | 1.66 | 59 | 21.411 | Y | Y |
| 8 | female | 42 | 1.60 | 49 | 19.141 | Y | Y |
| 9 | female | 45 | 1.68 | 60 | 21.259 | Y | Y |
| 10 | female | 46 | 1.68 | 62 | 21.967 |  | Y |
| 11 | female | 45 | 1.62 | 54 | 20.576 |  | Y |
| 12 | female | 43 | 1.65 | 63 | 23.140 |  | Y |
| 13 | female | 66 | 1.62 | 55 | 20.957 | Y | Y |
| 14 | female | 68 | 1.60 | 55 | 21.484 | Y | Y |
| 15 | female | 69 | 1.63 | 63 | 23.712 | Y | Y |
| 16 | female | 61 | 1.61 | 57 | 21.990 |  | Y |
| 17 | female | 61 | 1.65 | 65 | 23.875 |  | Y |
| 18 | female | 62 | 1.67 | 62 | 22.231 |  | Y |

**Table S1**. Clinic information of volunteers supplied SAT. SAT were used for morphological observation through SEM and examination of mRNA expression of DEGs through RT-qPCR.
