# Supplementary material for: New insights into aging-associated characteristics of female subcutaneous adipose tissue through integrative analysis of multi-omics data
Source: Bioengineered. 2022 Jan 9;13(2):2044–57. doi: 10.1080/21655979.2021.2020467 (PMC8973830; doi:10.1080/21655979.2021.2020467)
Supplement: Supplemental Material [file KBIE_A_2020467_SM1083.zip › supplementary/Table S2clean.docx]

| **Group** | **Score** | **Collagen curling** | **Collagen wrinkling** | **NSC integrity** | **NSC density** | **Bundle formation of collagen** | **Shrinking of the adipocytes** |
| --- | --- | --- | --- | --- | --- | --- | --- |
| **Sample 1** | **1** |  |  |  |  |  |  |
|  | **2** |  |  |  |  |  |  |
|  | **3** |  |  |  |  |  |  |
|  | **4** |  |  |  |  |  |  |
|  | **5** |  |  |  |  |  |  |
| **Sample 2** | **1** |  |  |  |  |  |  |
|  | **2** |  |  |  |  |  |  |
|  | **3** |  |  |  |  |  |  |
|  | **4** |  |  |  |  |  |  |
|  | **5** |  |  |  |  |  |  |
| **Sample 3** | **1** |  |  |  |  |  |  |
|  | **2** |  |  |  |  |  |  |
|  | **3** |  |  |  |  |  |  |
|  | **4** |  |  |  |  |  |  |
|  | **5** |  |  |  |  |  |  |
| **Sample 4** | **1** |  |  |  |  |  |  |
|  | **2** |  |  |  |  |  |  |
|  | **3** |  |  |  |  |  |  |
|  | **4** |  |  |  |  |  |  |
|  | **5** |  |  |  |  |  |  |
| **Sample 5** | **1** |  |  |  |  |  |  |
|  | **2** |  |  |  |  |  |  |
|  | **3** |  |  |  |  |  |  |
|  | **4** |  |  |  |  |  |  |
|  | **5** |  |  |  |  |  |  |
| **Sample 6** | **1** |  |  |  |  |  |  |
|  | **2** |  |  |  |  |  |  |
|  | **3** |  |  |  |  |  |  |
|  | **4** |  |  |  |  |  |  |
|  | **5** |  |  |  |  |  |  |
| **Sample 7** | **1** |  |  |  |  |  |  |
|  | **2** |  |  |  |  |  |  |
|  | **3** |  |  |  |  |  |  |
|  | **4** |  |  |  |  |  |  |
|  | **5** |  |  |  |  |  |  |
| **Sample 8** | **1** |  |  |  |  |  |  |
|  | **2** |  |  |  |  |  |  |
|  | **3** |  |  |  |  |  |  |
|  | **4** |  |  |  |  |  |  |
|  | **5** |  |  |  |  |  |  |
| **Sample 9** | **1** |  |  |  |  |  |  |
|  | **2** |  |  |  |  |  |  |
|  | **3** |  |  |  |  |  |  |
|  | **4** |  |  |  |  |  |  |
|  | **5** |  |  |  |  |  |  |

**Table S2.** Blinding measures were designed for the morphology of collagen and mature adipocytes. 1-5 were corresponding to *few*，*generally less*，*middle*，*generally more*，*many*. Sample 1-3 were from Youth group, 4-6 from Middle-aged group and 7-9 from Elderly group. 5 observers participated in scoring and the results in summary were shown as mean ± standard deviation. Abbreviation: NSC, net structure of collagen.
