# Supplementary material for: New insights into aging-associated characteristics of female subcutaneous adipose tissue through integrative analysis of multi-omics data
Source: Bioengineered. 2022 Jan 9;13(2):2044–57. doi: 10.1080/21655979.2021.2020467 (PMC8973830; doi:10.1080/21655979.2021.2020467)
Supplement: Supplemental Material [file KBIE_A_2020467_SM1083.zip › supplementary/Table S3clean.docx]

|  | **DEmRNA** | | **DEmiRNA** | | **DMR** | |
| --- | --- | --- | --- | --- | --- | --- |
|  | **Obese** | **Nonobese** | **Obese** | **Nonobese** | **Obese** | **Nonobese** |
| **Youth** | 3 | 6 | 3 | 6 | 3 | 6 |
| **Middle Age** | 11 | 9 | 11 | 9 | 11 | 9 |
| **Elder** | 3 | 5 | 3 | 5 | 3 | 5 |

**Table S3** The expression profiling data were obtained from 37 female, which were divided into groups according to ages and obese status.
