# Supplementary material for: New insights into aging-associated characteristics of female subcutaneous adipose tissue through integrative analysis of multi-omics data
Source: Bioengineered. 2022 Jan 9;13(2):2044–57. doi: 10.1080/21655979.2021.2020467 (PMC8973830; doi:10.1080/21655979.2021.2020467)
Supplement: Supplemental Material [file KBIE_A_2020467_SM1083.zip › supplementary/Table S4clean.docx]

| **Sample ID** | **Obese status** | **Gender** | **Age** | **Age Group** | **Methylation** | **mRNA** | **miRNA** |
| --- | --- | --- | --- | --- | --- | --- | --- |
| WAT-1 | Nonobese | Female | 23 | Youth | GSM611991 | GSM623796 | GSM625476 |
| WAT-2 | Nonobese | Female | 24 | Youth | GSM611972 | GSM623789 | GSM625505 |
| WAT-3 | Nonobese | Female | 24 | Youth | GSM611978 | GSM623798 | GSM625478 |
| WAT-4 | Nonobese | Female | 26 | Youth | GSM611975 | GSM623806 | GSM625513 |
| WAT-5 | Nonobese | Female | 26 | Youth | GSM612014 | GSM623816 | GSM625516 |
| WAT-6 | Nonobese | Female | 29 | Youth | GSM611996 | GSM623782 | GSM625502 |
| WAT-7 | Nonobese | Female | 41 | Middel Age | GSM611995 | GSM623776 | GSM625499 |
| WAT-8 | Nonobese | Female | 41 | Middel Age | GSM612012 | GSM623792 | GSM625475 |
| WAT-9 | Nonobese | Female | 42 | Middel Age | GSM611985 | GSM623803 | GSM625481 |
| WAT-10 | Nonobese | Female | 43 | Middel Age | GSM612005 | GSM623763 | GSM625462 |
| WAT-11 | Nonobese | Female | 44 | Middel Age | GSM611981 | GSM623774 | GSM625497 |
| WAT-12 | Nonobese | Female | 46 | Middel Age | GSM611980 | GSM623770 | GSM625495 |
| WAT-13 | Nonobese | Female | 46 | Middel Age | GSM611982 | GSM623775 | GSM625498 |
| WAT-14 | Nonobese | Female | 48 | Middel Age | GSM612013 | GSM623771 | GSM625496 |
| WAT-15 | Nonobese | Female | 49 | Middel Age | GSM611976 | GSM623766 | GSM625465 |
| WAT-16 | Nonobese | Female | 60 | Elder | GSM612000 | GSM623781 | GSM625472 |
| WAT-17 | Nonobese | Female | 61 | Elder | GSM612023 | GSM623778 | GSM625500 |
| WAT-18 | Nonobese | Female | 65 | Elder | GSM611999 | GSM623762 | GSM625463 |
| WAT-19 | Nonobese | Female | 68 | Elder | GSM612015 | GSM623761 | GSM625491 |
| WAT-20 | Nonobese | Female | 72 | Elder | GSM611971 | GSM623765 | GSM625493 |
| WAT-21 | Obese | Female | 27 | Youth | GSM611993 | GSM623784 | GSM625504 |
| WAT-22 | Obese | Female | 29 | Youth | GSM611990 | GSM623812 | GSM625488 |
| WAT-23 | Obese | Female | 30 | Youth | GSM612003 | GSM623787 | GSM625473 |
| WAT-24 | Obese | Female | 41 | Middel Age | GSM611974 | GSM623807 | GSM625484 |
| WAT-25 | Obese | Female | 41 | Middel Age | GSM612019 | GSM623808 | GSM625485 |
| WAT-26 | Obese | Female | 42 | Middel Age | GSM611992 | GSM623799 | GSM625511 |
| WAT-27 | Obese | Female | 42 | Middel Age | GSM611997 | GSM623805 | GSM625483 |
| WAT-28 | Obese | Female | 43 | Middel Age | GSM612025 | GSM623804 | GSM625482 |
| WAT-29 | Obese | Female | 44 | Middel Age | GSM612022 | GSM623794 | GSM625509 |
| WAT-30 | Obese | Female | 45 | Middel Age | GSM611987 | GSM623780 | GSM625501 |
| WAT-31 | Obese | Female | 45 | Middel Age | GSM612007 | GSM623790 | GSM625506 |
| WAT-32 | Obese | Female | 45 | Middel Age | GSM612017 | GSM623802 | GSM625512 |
| WAT-33 | Obese | Female | 47 | Middel Age | GSM611988 | GSM623791 | GSM625507 |
| WAT-34 | Obese | Female | 48 | Middel Age | GSM611977 | GSM623800 | GSM625479 |
| WAT-35 | Obese | Female | 63 | Elder | GSM612024 | GSM623779 | GSM625471 |
| WAT-36 | Obese | Female | 64 | Elder | GSM612026 | GSM623777 | GSM625470 |
| WAT-37 | Obese | Female | 66 | Elder | GSM612004 | GSM623767 | GSM625466 |

**Table S4.** The groups and sources of each sample involved in the multi-omics analysis.
