# Supplementary material for: New insights into aging-associated characteristics of female subcutaneous adipose tissue through integrative analysis of multi-omics data
Source: Bioengineered. 2022 Jan 9;13(2):2044–57. doi: 10.1080/21655979.2021.2020467 (PMC8973830; doi:10.1080/21655979.2021.2020467)
Supplement: Supplemental Material [file KBIE_A_2020467_SM1083.zip › supplementary/Table S5clean.docx]

| WGCNA Module | turquoise | blue | brown | yellow | green | red |
| --- | --- | --- | --- | --- | --- | --- |
| Gene Numbers | 526 | 509 | 505 | 411 | 407 | 405 |
| WGCNA Module | **black** | **pink** | **magenta** | **purple** | **greenyellow** | **out of modules** |
| Gene Numbers | 387 | 294 | 257 | 245 | 192 | 422 |

**Table S5** The numbers of co-expression DEGs in each gene module based on WGCNA for non-obese female.
