# Supplementary material for: New insights into aging-associated characteristics of female subcutaneous adipose tissue through integrative analysis of multi-omics data
Source: Bioengineered. 2022 Jan 9;13(2):2044–57. doi: 10.1080/21655979.2021.2020467 (PMC8973830; doi:10.1080/21655979.2021.2020467)
Supplement: Supplemental Material [file KBIE_A_2020467_SM1083.zip › supplementary/Table S6clean.docx]

| WGCNA Module | black | blue | brown | green |
| --- | --- | --- | --- | --- |
| Gene Numbers | 38 | 80 | 43 | 39 |
| WGCNA Module | **yellow** | **red** | **turquoise** | **out of modules** |
| Gene Numbers | 40 | 38 | 261 | 4021 |

**Table S6.** The numbers of differential co-expression genes in each gene module based on WGCNA between obese and non-obese female.
