# Supplementary figures and images for: New insights into aging-associated characteristics of female subcutaneous adipose tissue through integrative analysis of multi-omics data
Source: Bioengineered. 2022 Jan 9;13(2):2044–57. doi: 10.1080/21655979.2021.2020467 (PMC8973830; doi:10.1080/21655979.2021.2020467)

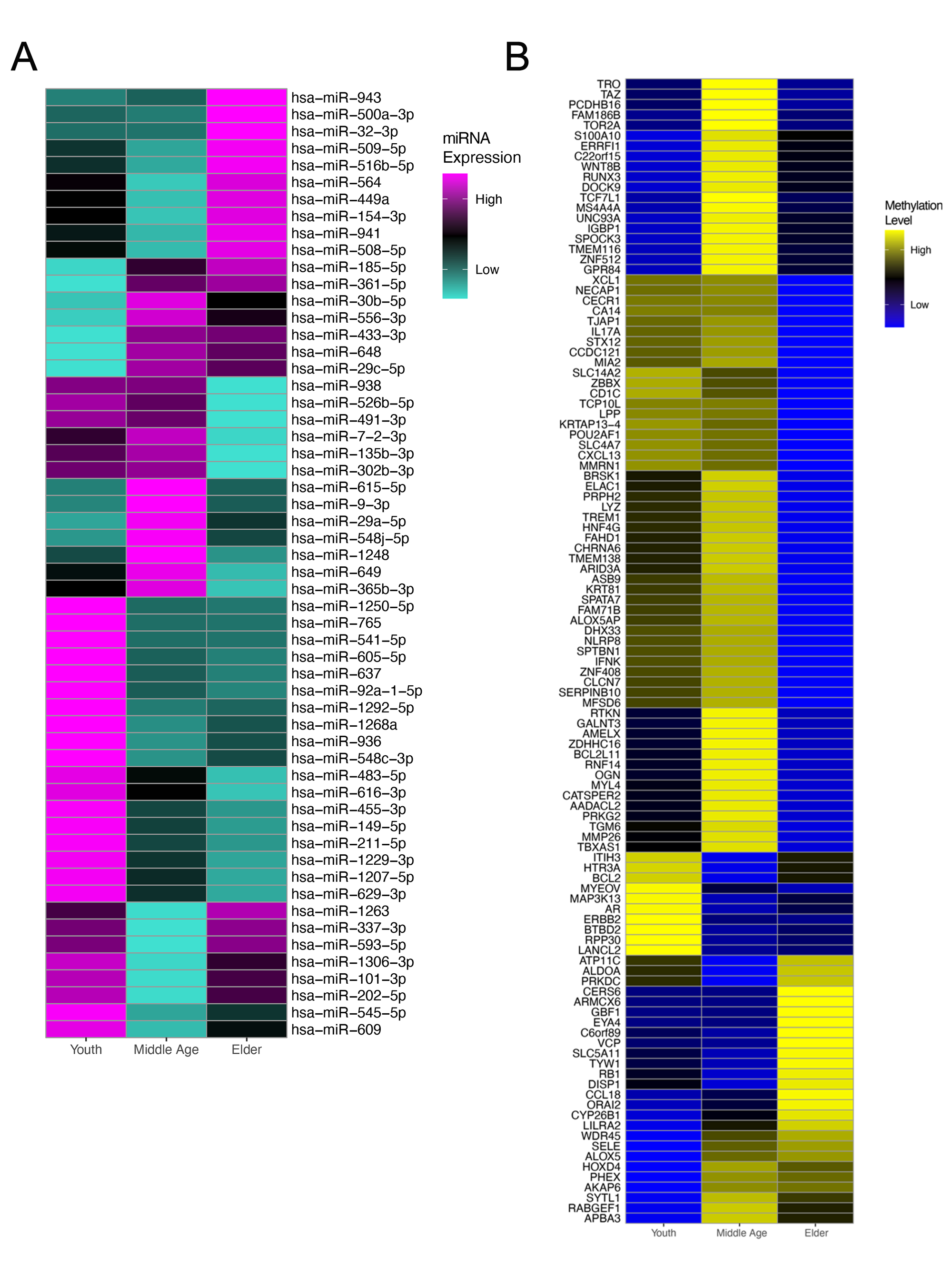

Supplement: Supplemental Material [file KBIE_A_2020467_SM1083.zip › supplementary/Figure S1.tif]

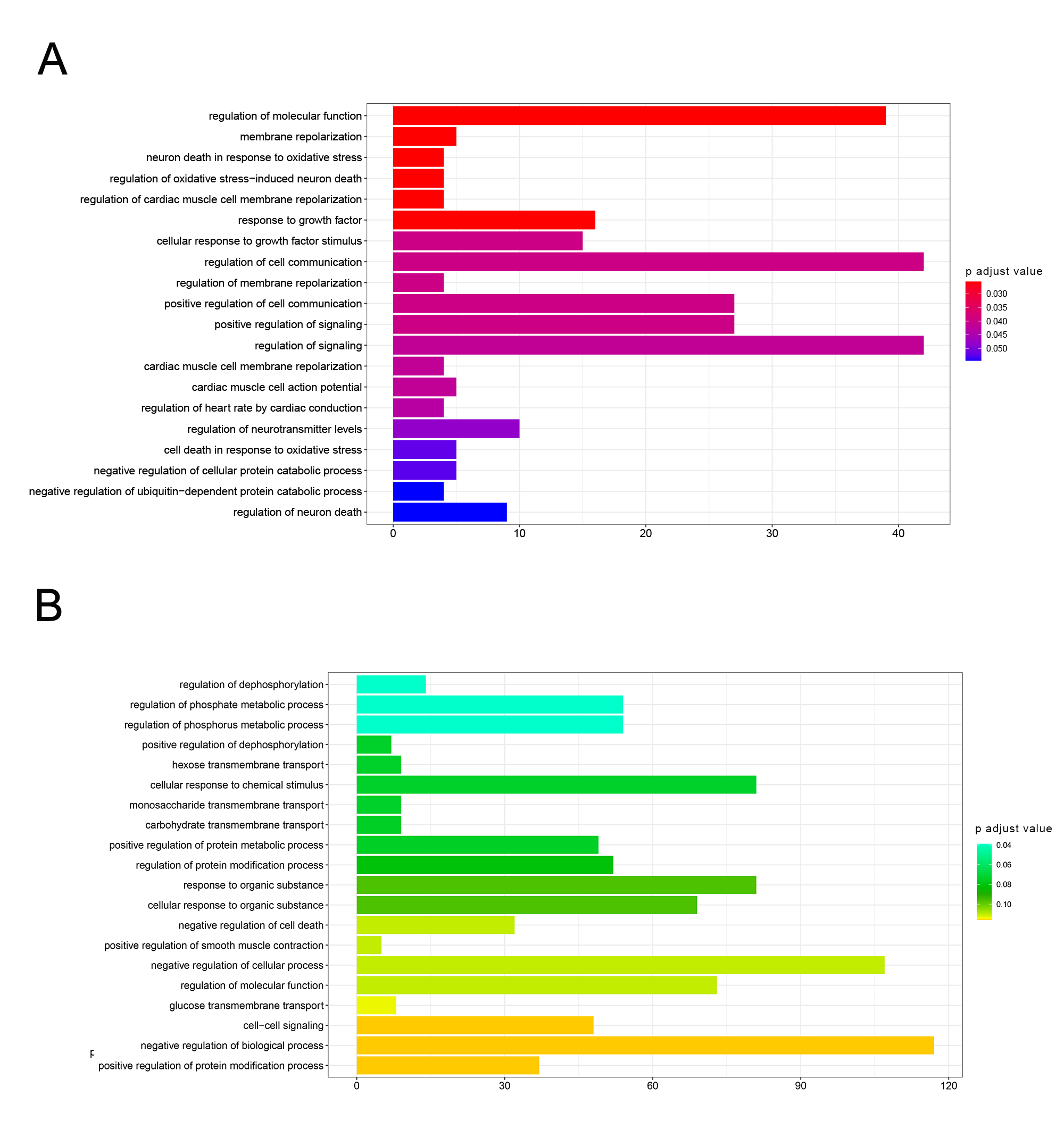

Supplement: Supplemental Material [file KBIE_A_2020467_SM1083.zip › supplementary/Figure S2.tif]

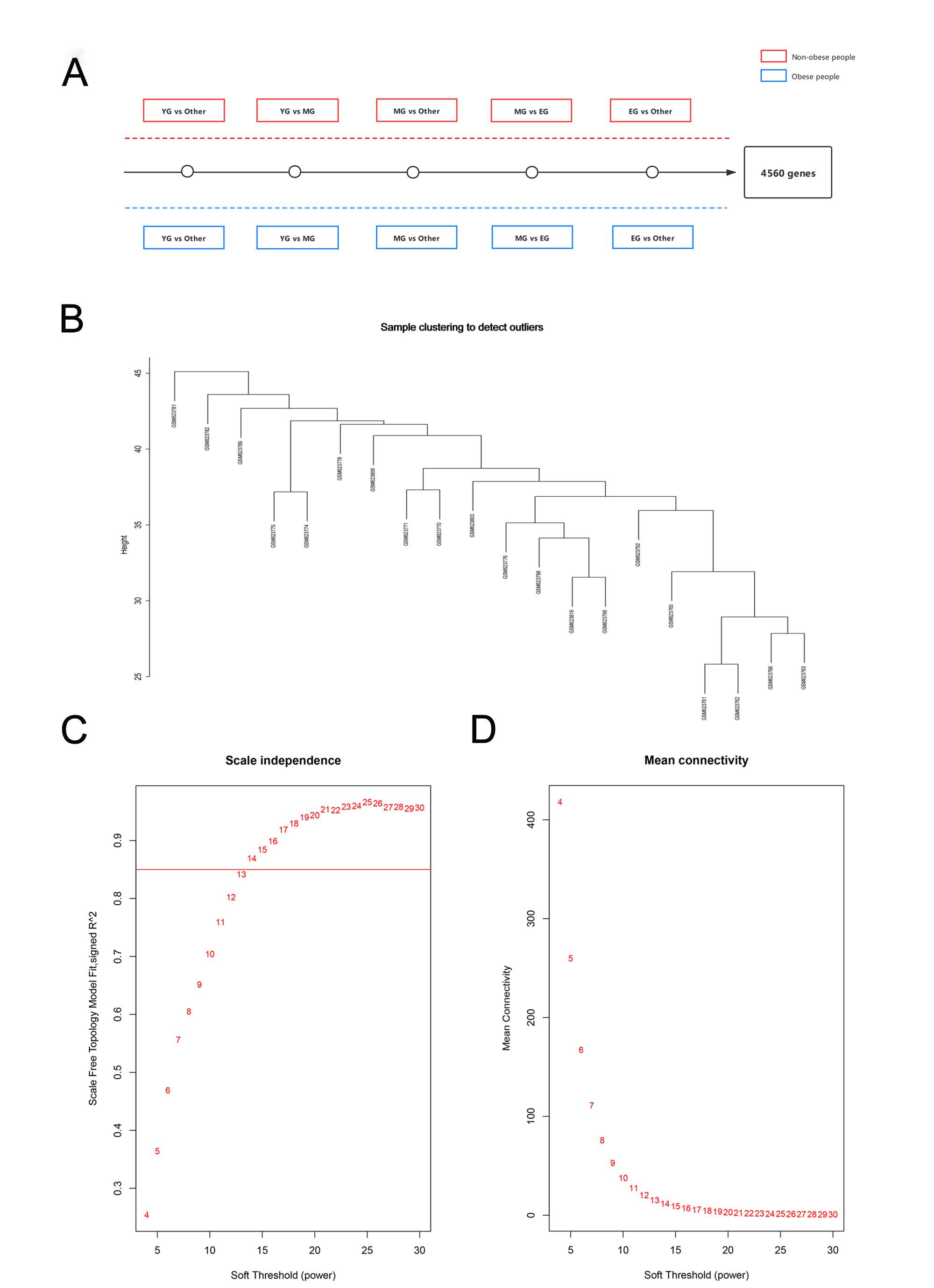

Supplement: Supplemental Material [file KBIE_A_2020467_SM1083.zip › supplementary/Figure S3.tif]

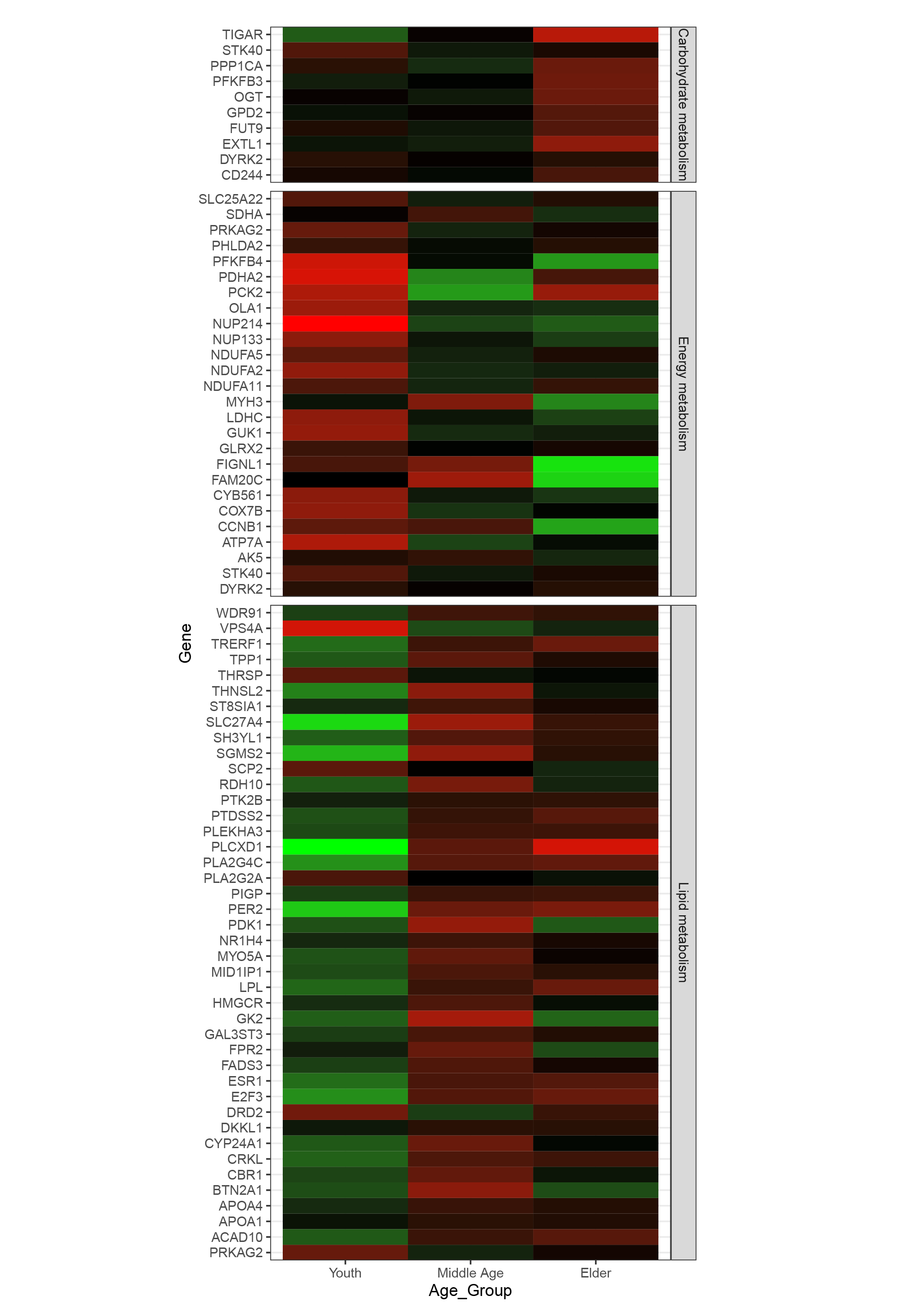

Supplement: Supplemental Material [file KBIE_A_2020467_SM1083.zip › supplementary/Figure S4.tif]
